# White Adipocyte Plasticity in Physiology and Disease

**DOI:** 10.3390/cells8121507

**Published:** 2019-11-25

**Authors:** Ewa Bielczyk-Maczynska

**Affiliations:** Department of Chemical and Systems Biology, Stanford University, Stanford, CA 94305, USA; ewabm@stanford.edu

**Keywords:** cell plasticity, adipocytes, fat, cell dedifferentiation, cell transdifferentiation, cell differentiation

## Abstract

Cellular plasticity is a transformation of a terminally differentiated cell into another cell type, which has been long known to occur in disease and regeneration. However, white adipocytes (fat cells) have only recently been observed to undergo different types of cellular plasticity. Adipocyte transdifferentiation into myofibroblasts and cancer-associated fibroblasts occurs in fibrosis and cancer, respectively. On the other hand, reversible adipocyte dedifferentiation into adipocyte progenitor cells (preadipocytes) has been demonstrated in mammary gland and in dermal adipose tissue. Here we discuss the research on adipocyte plasticity, including the experimental approaches that allowed to detect and study it, the current state of the knowledge, major research questions which remain to be addressed, and the advances required to stimulate adipocyte plasticity research. In the future, the knowledge of the molecular mechanisms of adipocyte plasticity can be utilized both to prevent adipocyte plasticity in disease and to stimulate it for use in regenerative medicine.

## 1. Introduction

**Figure 1:**
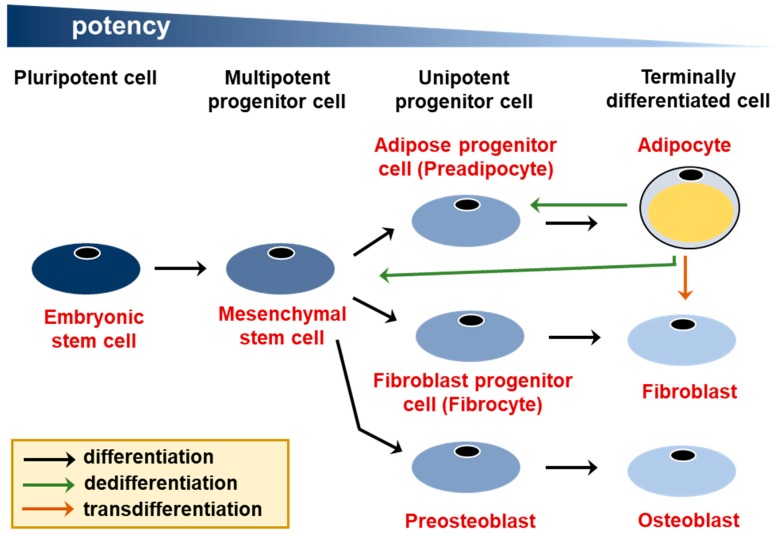
Two types of cellular plasticity—dedifferentiation and transdifferentiation. Adipocytes are derived from multipotent mesenchymal stem cells, which can also give rise to fibroblasts, osteoblasts, and other cell types, such as muscle cells and chondrocytes (not shown). Adipocyte dedifferentiation into a preadipocyte and a mesenchymal stem cell, as well as transdifferentiation into a fibroblast are indicated with arrows.

In the traditional view of cell differentiation, cells follow a differentiation trajectory in discrete developmental stages, beginning with stem cells and culminating with a terminally differentiated state [1]. The terminally differentiated state is thought to be permanent as the cells can no longer transfer into other cell fates. However, in many systems the phenomenon of cellular plasticity, which is a transformation of a cellular phenotype beginning with a terminally differentiated cell, has been described not only in pathologies like cancer, but also during physiological processes [2]. While many categories of cellular plasticity have been proposed, here we will distinguish between dedifferentiation, which involves a differentiated cell reverting to a cell with a greater developmental potential, such as a stem or progenitor cell, and transdifferentiation, in which a differentiated cell transfers into another differentiated cell type (Figure 1). Transdifferentiation may involve an intermediate dedifferentiation step [3]. 

Some types of cellular plasticity, for example, the regeneration of lens in newt [4], have been recognized for a long time. However, recent advances in experimental models which allow to unequivocally determine the developmental source of a particular cell have led to renewed interest in cellular plasticity [3], especially because of the implications for regenerative medicine. In addition, novel experimental approaches allow to induce cellular plasticity through reprogramming of differentiated cells—in the most prominent example, to obtain induced pluripotent stem cells (iPCSs) from fibroblasts [5].

Adipose (fat) tissue is a major endocrine organ whose primary function is the long-term storage of energy in the form of lipids. It is distributed across multiple depots in the body and is comprised of fat cells (adipocytes), adipose progenitor cells, and other non-adipogenic cell types, including fibroblasts, endothelial cells, and neurons. There are two major types of adipose tissue present in mammals. White adipocyte tissue (WAT) contains large adipocytes filled with single large lipid droplets and its primary function is long-term energy storage and insulation. In contrast, brown adipose tissue (BAT) is comprised of adipocytes with multiple small lipid droplets capable of producing heat (thermogenesis). Adipose (fat) tissue has long been viewed as relatively dispensable, given that its primary function—long-term storage of energy—seems excessive in the age of permanent calorie excess. Even though we currently understand that fat tissue has many additional functions, for example, as a major endocrine organ [6], the fact that its volume can drastically differ between individuals suggests that there is a large reserve of cells capable of replacing lost fat cells (adipocytes). In addition, fat tissue is routinely removed during cosmetic procedures and finding ways to put these cells to use for the purpose of regenerative medicine is worth exploring. Recent insights into adipocyte plasticity in physiology and disease, discussed here, shed light on the alternative cellular states that adipocytes can assume. Even though there is ample evidence for the presence of BAT in humans [7,8], BAT constitutes, by far, the lower percentage of whole-body adipose tissue in adult humans compared to mice. For this reason, this review primarily focuses on the plasticity of white adipose tissue. 

In general, cellular plasticity either follows the reverse of differentiation trajectory or occurs between closely related lineages. For example, in the pancreas different types of conversions have been observed between acinar cells, α cells, β cells, and δ cells, all of which are derived from a common multipotent progenitor cell [9]. In case of fat tissue, lineage-committed adipose progenitor cells (preadipocytes) arise from mesenchymal stem cells, which can also give rise to chondrocytes, osteoblasts, and myocytes [10]. Below, we review different types of adipocyte plasticity which have been described to date. For review on cellular plasticity in other tissues see [2,3].

## 2. The Extent of White Adipocyte Plasticity in Physiological Processes and in Disease

### 2.1. White Adipocyte Beiging

Brown adipocytes can produce heat (thermogenesis) through uncoupling protein-1 (UCP1) which dissociates electron transport from ATP production in mitochondria [11]. Prolonged cold exposure or direct stimulation with β-adrenergic agonists leads to the appearance of cells with the phenotype of brown adipocytes within classical WAT depots (called beige or brite adipocytes). This phenomenon, termed WAT beiging, has sparked a lot of interest given that in humans the presence of beige adipocytes is correlated with a metabolically healthy phenotype [12,13,14]. Furthermore, genetic manipulations resulting in the appearance of beige adipocytes within WAT protect against the development of obesity in mice [15]. Both direct transdifferentiation of white adipocytes [16] and de novo differentiation of beige adipocytes from progenitor cells [17,18] have been implicated in the appearance of beige adipocytes. However, the process of white fat beiging is reversible upon the removal of the beiging stimulus [16], suggesting that beige adipocytes represent a transient cell state rather than a stable change of cell identity. The understanding of the beiging process at the molecular level and development of methods to induce and maintain beige adipocytes are a rapidly growing field of research and are comprehensively reviewed elsewhere [11].

### 2.2. Adipocyte Dedifferentiation in Mammary Gland and Skin

The mammary gland, which contains a high percentage of white adipocytes, undergoes dramatic remodeling firstly during lactation and later again during mammary gland involution. In lactation, fat tissue gives way to the expanding mammary epithelium, accompanied by dedifferentiation of adipocytes to preadipocytes. The dedifferentiated preadipocytes can re-enter the cell cycle and proliferate, as well as re-differentiate into adipocytes during the involution of the mammary gland [19]. 

In addition to dedifferentiation into preadipocytes, previous research indicated that adipocytes in the mammary gland can transdifferentiate into mammary epithelial cells [20,21,22], while more recent studies contradict this possibility [19,23]. However, recently it was found that the platelet-derived growth factor receptor A (Pdgfrα) positive adipocyte progenitor cells, present in the mammary gland stroma, can give rise to epithelial cells of the mammary gland [24], which shows another unexpected type of cellular plasticity in the mammary gland.

**Figure 2:**
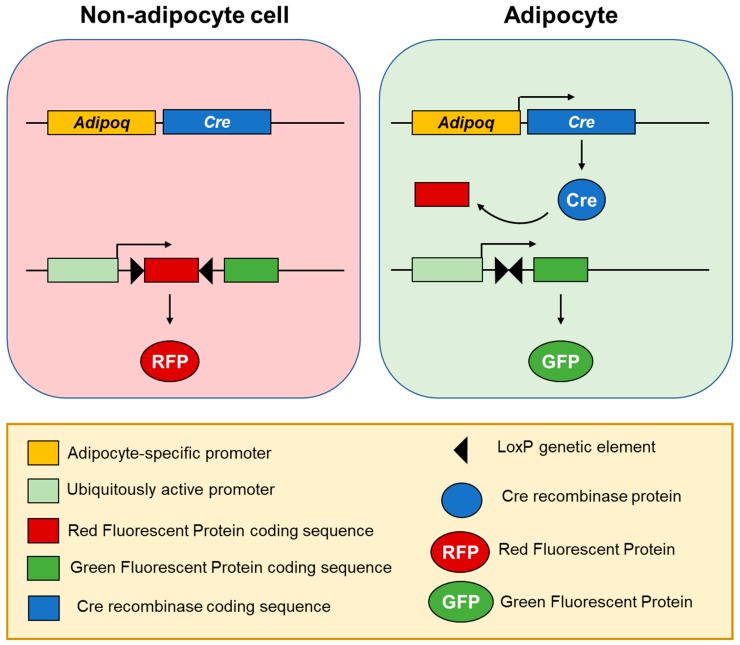
Adipocyte lineage tracing using fluorescent labelling [30]. In a transgenic mouse model, Cre recombinase is expressed under an adipocyte-specific promoter, such as *Adipoq*, leading to its specific expression in differentiated adipocytes, but not adipose progenitor cells nor any other cell types. Another transgene drives constant expression of a fluorescent protein: A Red Fluorescent Protein (RFP) (by default) or Green Fluorescent Protein (GFP) after Cre recombinase mediates excision of the genetic element encoding RFP. Therefore, cell fluorescence switches from red to green only in adipocytes and this genetic change is carried over to all cells derived from them, allowing for adipocyte lineage tracing in the studies on adipocyte plasticity.

Discrepancies between the results of earlier and more recent studies on the possibility of adipocyte-epithelial cell transdifferentiation are most likely due to the differences between mouse genetic models used for the tracing of adipocyte lineage. Lineage tracing is an experimental approach in which irreversible genetic change occurs only in cells of a specific cell type (lineage), leading to the permanent expression of a marker protein in particular types of marker-expressing cells and all cells derived from them (Figure 2). Therefore, this technique can be applied to permanently mark adipocytes and track their eventual fate in the body. However, lineage tracing is limited by the quality of the used genetic tools, as it depends on the use of a lineage-specific marker gene. In the early studies, the expression of the *aP2* gene was widely used to lineage-trace adipocytes [25]. However, in addition to most adipocytes, *aP2* is expressed in a fraction of undifferentiated adipogenic cells, as well as in macrophages which are present in fat tissue [26]. A similarly unspecific pattern of gene expression was observed for other candidate adipocyte markers—*Retn* [27] and *Cdh5* [28]. The development of lineage tracing based on the gene promoter of the adipocyte-specific cytokine adiponectin (*Adipoq:Cre* mouse models) led to the increased reliability of the adipocyte lineage tracing [29,30]. The introduction of the *Adipoq:Cre* lineage tracing challenged not only the possibility of the adipocyte-epithelial cell transdifferentiation in the mammary gland, but also of endothelial and myeloid sources of adipocytes [31,32,33]. However, it is worth pointing out that the possible cellular sources of adipocytes, especially for the ones arising after wounding, are still very much debated. Novel experimental techniques, such as single-cell RNA sequencing, support the possibility of a myeloid origin for some adipocytes [32].

**Figure 3:**
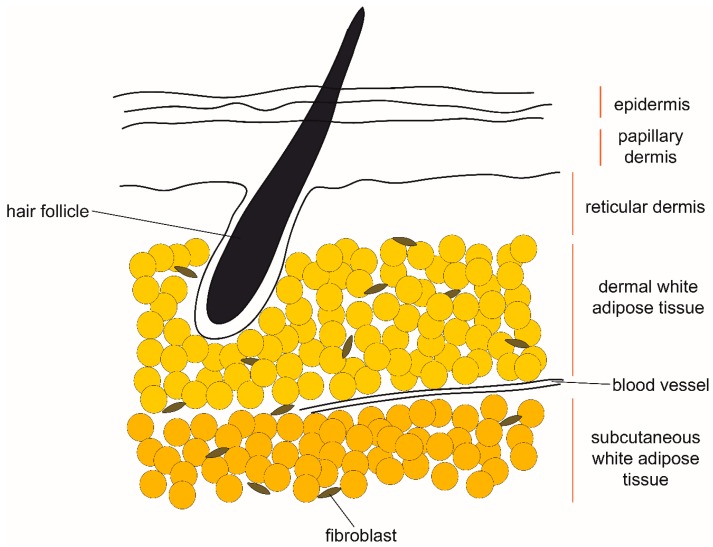
The anatomical location of dermal and subcutaneous white adipose tissue in human skin. Proposed sources of signals which induce remodeling of dermal white adipose tissue include hair follicles and fibroblasts, as well as hormones circulating in blood.

Dermal adipose tissue is located within the skin and is molecularly distinct from the adjacent subcutaneous adipose tissue (Figure 3). In humans there is no clear anatomical boundary between the dermal and subcutaneous adipose tissue layers, while in mice they are separated by a striated muscle called panniculus carnosus [34]. There are clear histological and metabolic differences between the two layers of fat associated with skin [35,36]. Dermal adipose tissue has been indicated in such processes as immune response, thermoregulation, and wound healing [37] and it exhibits pronounced changes in size in synchrony with hair cycling. It was recently demonstrated that, similarly to the process observed in the mammary gland, reversible dedifferentiation of pre-existing adipocytes largely contributes to the observed changes in dermal adipose tissue size over time [38]. The parallels between adipocyte dedifferentiation in skin and mammary gland suggest that the process is regulated by signals from the adjacent cutaneous structures–mammary glands and hair follicles, respectively. Signaling from hair follicles through bone morphogenetic protein (BMP), insulin-like growth factor (IGF) and Sonic hedgehog (Shh) ligands is already known to induce adipogenesis in the adjacent dermal adipose tissue [39,40]. On the other hand, the dedifferentiation ability could also be cell-intrinsic and stem from separate differentiation origins of various fat depots, such as in the case of dermal and subcutaneous adipocytes [41].

### 2.3. Adipocyte Transdiferentiation into Myofibroblasts and Cancer-Associated Fibroblasts

Fibrosis is a highly progressive disease characterized by the hardening of tissue, which is attributed to excessive deposition of extracellular matrix components, such as collagens. The progress of fibrosis is largely driven by myofibroblasts, cells which exhibit intermediate features between smooth muscle cells and fibroblasts [42]. Under physiological conditions myofibroblasts differentiate from tissue-resident fibroblasts following tissue injury. The myofibroblasts mediate wound healing and undergo apoptosis afterwards [43]. Fibrosis develops in response to repeated or chronic injury, when the presence of activated myofibroblasts in the tissue becomes permanent [44]. It is now argued that myofibroblasts are not a specific cell type but rather a cell state that can be assumed by various cell types, including resident mesenchymal cells, epithelial cells, and endothelial cells [44,45]. 

Recently through the use of adiponectin-dependent lineage tracing it was shown that myofibroblasts can develop from intradermal adipocytes in a mouse model of dermal fibrosis [46]. Interestingly, there is an indication that the conversion from adipocytes to myofibroblasts in the skin may be reversible: a transition from myofibroblasts to adipocytes has been independently reported in skin wound healing in proximity to hair follicles [47]. 

Although the adipocyte-myofibroblast transition has only been observed in dermal fibrosis so far, certain cells which can give rise to myofibroblasts in other tissues share molecular characteristics with adipocytes. In a mouse model of lung fibrosis the source cell for myofibroblasts are lipogenic fibroblasts, which are interstitial fibroblasts containing lipid droplets [48]. In experimental models lipogenic fibroblasts can also differentiate into myofibroblasts in response to hyperoxia [49] or nicotine exposure [50]. Similarly, the well-described source of myofibroblasts in liver fibrosis are hepatic stellate cells [51]—liver-specific mesenchymal cells which store vitamin A in their lipid droplets [52]. The molecular similarities between adipocytes, lipogenic fibroblast in the lung, and hepatic stellate cells in the liver pose a question: is there a broader mechanism linking a lipogenic phenotype with the potential for cell transdifferentiation into myofibroblasts? A possible switch-like mechanism between the myofibroblast and the lipogenic state is supported by the antifibrotic role of the key adipogenic transcription factor peroxisome proliferator-activated receptor-γ (PPARγ) [48,53,54].

In addition to myofibroblasts, adipocytes can transdifferentiate into cancer-associated fibroblasts (CAFs), which, phenotypically, closely resemble myofibroblasts [55]. A significant fraction of solid tumors comprise of noncancerous cells, including stromal cells. CAFs are the majority of the cancer-associated stromal cells present in breast cancers and other solid tumors. They play a major role in promoting tumor progression and metastasis [56]. The adipocyte-CAF transdifferentiation, observed in breast cancer mouse models [55], could potentially explain fat tissue disappearance during progression of tumors located in proximity to fat tissue, such as in breast and ovarian cancer, in addition to the mechanism of lipid transfer from adipocytes to cancer cells, which has been described in ovarian cancer [57]. 

Altogether, the conversion of adipocytes to myofibroblasts and CAFs likely represents a transition to a cellular state which under physiological conditions is temporary. However, the altered microenvironment of the fat tissue which initially likely stimulates the transdifferentiation may also be responsible for the long-term maintenance of myofibroblasts and CAFs, leading to a permanent cell state change.

### 2.4. Adipocyte Dedifferentiation in Liposarcomas

Liposarcomas (LPSs) are the most common type of soft-tissue cancer. Their categorization is based on cell morphology, and distinguishes between well-differentiated liposarcoma (WDLPS), dedifferentiated liposarcoma (DDLPS), myxoid/round cell liposarcoma (MLPS), and pleomorphic liposarcoma (PLPS) [58]. The cellular origin of LPSs in humans is not known. However, in mouse models overexpression of the constitutively active form of Notch1 in mature adipocytes leads to development of solid tumors which closely resemble LPSs. The tumor cells show strong upregulation of preadipocyte marker genes with concomitant downregulation of adipocyte marker genes, indicating dedifferentiation [59]. 

Adipocyte dedifferentiation in the pathogenesis of human liposarcomas is supported by the fact that methylation of the gene *CEBPA*, which encodes one of the key adipogenic transcription factors CCAAT/enhancer binding protein α (C/EBPα), is observed in 24% of DDLPS patients [60]. Interestingly, WDLPS/DDLPS cells can be induced to differentiate into adipocytes by the chemical stimulation routinely used to induce adipogenesis in adipocyte progenitor models in vitro [61]. It is also known that the adipogenic master regulator, transcription factor PPARγ, can revert the DDLPS phenotype subtype to a WDLPS-like [62,63], which is associated with better health outcomes. Altogether, it appears that the more dedifferentiated the phenotype of the liposarcoma, the worse the clinical outcome and the risk of recurrence after surgical resection [64]. The conserved responsiveness of liposarcoma to adipogenic differentiation stimuli suggests that differentiation therapy can find clinical use, given only 30% 5-year survival of DDLPS patients [65].

## 3. Experimentally Induced White Adipocyte Plasticity

Given that cellular plasticity allows for cells to transfer into ontogenically close lineages, one would expect that white adipocytes may be able to give rise to other mesenchymal cell types, such as muscle cells or osteoblasts, which would make them an important potential cellular source for regeneration medicine, especially since large numbers of adipocytes can be obtained during a biopsy [66]. 

Due to their large size and low density because of their high lipid content, primary adipocytes are challenging to culture in vitro. However, adipocytes can be cultured using a so-called ceiling culture method, in which mature adipocytes adhere to the top surface of a tissue culture flask which is completely filled with culture media [67]. After approximately eight days of culture, a large number of proliferating fibroblast-like cells appear [68]. These cells, referred to as dedifferentiated adipocytes (DFAT cells), express markers of embryonic stem cells and can differentiate not only into adipocytes but also into osteocytes, chondrocytes, and cardiomyocytes, indicating that they are multipotent [68,69,70,71,72]. However, only approximately half of cells in adipose tissue are adipocytes, with many other cell populations present. DFAT cells may derive from non-adipocyte populations contaminating the floating fraction, for example from the multipotent mesenchymal stem cells (MSCs) [73]. Since the majority of DFAT-related research uses human primary adipocytes as the model, it is not possible to conduct lineage tracing experiments to confirm the adipocyte origin of the DFAT cells. Therefore, the unequivocal determination of DFAT cellular sources remains to be tested using lineage tracking experiments in rodents or other relevant models. Whether DFAT cells are a product of white adipocyte plasticity or not, their use for regenerative purposes is an exciting avenue of research [74].

## 4. Molecular Mechanisms of White Adipocyte Plasticity

The transcriptional regulation of adipocyte differentiation and adipocyte function has been relatively well described compared to the mechanisms which maintain adipocyte state and prevent adipocyte plasticity under normal conditions. 

Adipogenesis is driven by a coordinated function of many transcription factors, including key early regulators CCAAT/enhancer binding proteins (C/EBPs) and the master regulator of adipogenesis PPARγ [75,76]. PPARγ is necessary and sufficient to induce adipogenesis in a fibroblast cell line [77]. In adipocytes PPARγ and C/EBPs drive the expression of many genes which are responsible for adipocyte cellular functions, such as glucose uptake and lipid metabolism [78]. However, experimental downregulation of PPARγ in adipocytes in vitro has a minimal impact on cell morphology [79]. Only when coupled with the overexpression of transcription factor GATA2, the PPARγ downregulation causes gene expression reversal to a more preadipocyte-like state, although it is not clear whether these cells can re-enter the cell cycle and proliferate [80]. On the other hand, the antifibrotic effects of various PPARγ agonists [53] suggest that reinforcing the adipocyte state may prevent the adipocyte-myofibroblast transdifferentiation. Altogether these findings suggest that PPARγ inhibition or downregulation is required, but not sufficient for adipocyte plasticity, however this hypothesis remains to be directly tested.

**Figure 4:**
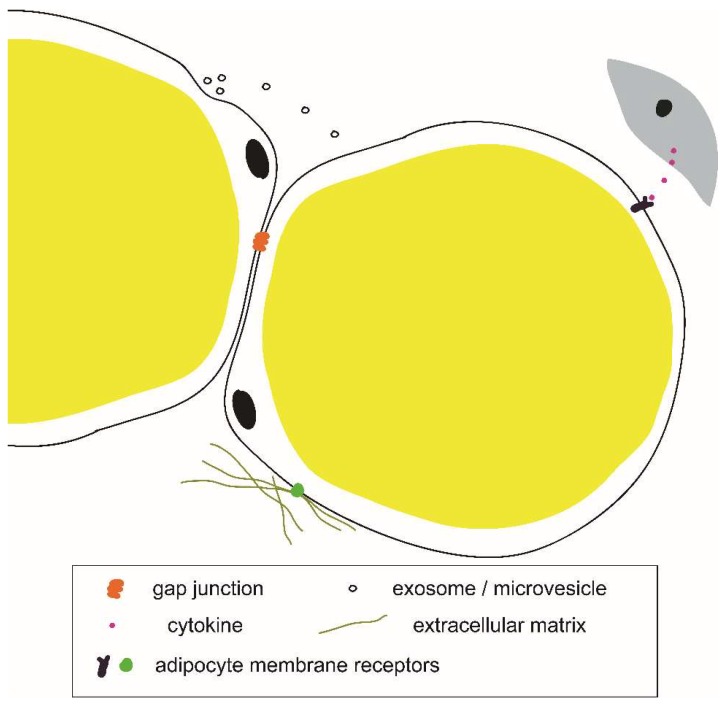
Example components of an adipocyte microenvironmental niche.

The signals inducing adipocyte plasticity in various physiological and disease processes are similarly not well understood. Several cytokines and secretory proteins have been implicated in driving adipocyte plasticity (Table 1). Properties of the local microenvironment, for example, the protein composition of the extracellular matrix, are known to affect adipocyte gene expression [81], adipose tissue growth, and remodeling [82,83]. In addition to cytokines and secretory proteins, there are other modes of intercellular communication which may be involved in white adipocyte plasticity, including exosomes and microvesicles [84,85], and direct cell-to-cell communication through gap junctions [86] (Figure 4). The cellular source of the adipocyte plasticity-inducing signals may be the neighboring cells. For example, breast cancer cells were shown to induce the loss of adipocyte phenotype in co-cultured 3T3-L1 adipocytes in vitro [55,56]. Interestingly, adipocytes themselves may produce certain plasticity-inducing signals, such as Fizz1 [87] and TGF-β [88]. Determining the molecular mechanisms of adipocyte plasticity is a key to the identification of novel drug targets and will inform therapies against cancer and fibrosis.

## 5. The Advances Required to Understand the Mechanisms of Adipocyte Plasticity

The development of lineage tracing tools greatly advanced our ability to detect adipocyte plasticity in experimental mouse models. However, a certain level of heterogeneity exists between different fat depots in the body, both in terms of gene expression [94] and cytokine secretion profiles [95]. Would adipocytes from different depots react similarly to the plasticity-inducing stimuli? For example, in the case of adipocyte dedifferentiation during lactation, does it occur in response to local signals in the mammary gland or does it occur in response to global hormonal changes to which only mammary gland adipocytes are responsive? These sorts of questions may be addressed by fat transplants between different depots, which is an established technique [96]. 

On the other hand, significant molecular differences between adipocytes within a single fat depot may also exist and affect a cell’s propensity for plasticity. The intra-depot adipocyte heterogeneity could be addressed by single-cell-omics approaches, which have been applied to uncover the heterogeneity of many other tissues and cell types [97,98]. Due to the extraordinarily large cell size and high lipid content of primary adipocytes, major technical difficulties are associated with using standard single-cell-omics approaches on primary adipocytes. Advancements towards overcoming these limitations in the future will allow us to study the extent of adipocyte heterogeneity within fat depots, in addition to the previously uncovered bulk differences between different depots.

## 6. Summary and Conclusions

In recent years different research groups elegantly demonstrated that adipocytes undergo plasticity. The research was greatly aided by the development of experimental tools which determine whether a particular cell in vivo originated from a mature adipocyte. However, our current knowledge of the extent of adipocyte plasticity is based on isolated findings in different disease models, rather than on a systematic identification of all possible cell types which adipocytes can transdifferentiate into. 

One outstanding mystery regarding adipocyte transdifferentiation is the ability of adipocytes to convert into other closely related mesodermal cell types such as osteoblasts or chondrocytes. Myofibroblasts, although usually derived from fibroblasts, are thought to represent a cell state rather than a true cell lineage in the traditional sense. On the other hand, DFAT cells, which show multipotent potential both in vitro and in vivo, have not been proven to derive from differentiated adipocytes using the golden standard of lineage tracing. 

Another unresolved question is the identity of plasticity-inducing molecular signals. Adipocyte transdifferentiation, which currently seems to occur uniquely in disease conditions, such as fibrosis and cancer, is mediated by the microenvironmental cues, such as cytokines [55]. That is most likely also true about adipocyte dedifferentiation in the context of lactation [19], but the possible microenvironmental signals which drive this process are currently not known. 

In summary, recent findings indicate that adipocyte plasticity plays a role in several physiological and pathological processes. Understanding the precise mechanisms of adipocyte trans- and dedifferentiation will enable novel approaches for the treatment of diseases, such as cancer and fibrosis. It will also clarify whether adipocytes can find use in regenerative medicine.

## Figures and Tables

**Table 1:** Cytokines and secretory proteins implicated in driving white adipocyte plasticity.

Plasticity-Inducing Signal	Described Process	Reference
Fizz1	Dedifferentiation of 3T3-L1 adipocytes in vitro by Fizz1	[87]
TGF-β	Conversion between adipogenic and lipogenic fibroblasts in the lung	[48]
TNF-α	Loss of adipocyte gene expression in 3T3-L1 adipocytes by a TNF-α treatment	[89,90]
Wnt10b	Progressive dermal fibrosis and the loss of adipose tissue in the skin of mice with an overexpression of Wnt10b	[91]
Wnt3a	Dedifferentiation of 3T3-L1 adipocytes in vitro by Wnt3a	[92]
Wnt5a	Dedifferentiation of 3T3-L1 adipocytes induced by the co-culture of pancreatic cancer MiaPaCa2 cells	[93]
